# Radiology Report Annotation Using Generative Large Language Models: Comparative Analysis

**DOI:** 10.1155/ijbi/5019035

**Published:** 2025-01-06

**Authors:** Bayan Altalla', Ashraf Ahmad, Layla Bitar, Mohammed Al-Bssol, Amal Al Omari, Iyad Sultan

**Affiliations:** ^1^Office of Scientific Affairs and Research, King Hussein Cancer Center, Amman, Jordan; ^2^School of Computing Sciences, Princess Sumaya University for Technology, Amman, Jordan; ^3^Artificial Intelligence and Data Innovation Office, King Hussein Cancer Center, Amman, Jordan

**Keywords:** GPT-4, in-context learning (ICL), large language models (LLMs), medical documentation automation, prompt engineering, radiology report annotation, retrieval-augmented generation (RAG)

## Abstract

Recent advancements in large language models (LLMs), particularly GPT-3.5 and GPT-4, have sparked significant interest in their application within the medical field. This research offers a detailed comparative analysis of the abilities of GPT-3.5 and GPT-4 in the context of annotating radiology reports and generating impressions from chest computed tomography (CT) scans. The primary objective is to use these models to assist healthcare professionals in handling routine documentation tasks. Employing methods such as in-context learning (ICL) and retrieval-augmented generation (RAG), the study focused on generating impression sections from radiological findings. Comprehensive evaluation was applied using a variety of metrics, including recall-oriented understudy for gisting evaluation (ROUGE) for n-gram analysis, Instructor Similarity for contextual similarity, and BERTScore for semantic similarity, to assess the performance of these models.

The study shows distinct performance differences between GPT-3.5 and GPT-4 across both zero-shot and few-shot learning scenarios. It was observed that certain prompts significantly influenced the performance outcomes, with specific prompts leading to more accurate impressions. The RAG method achieved a superior BERTScore of 0.92, showcasing its ability to generate semantically rich and contextually accurate impressions. In contrast, GPT-3.5 and GPT-4 excel in preserving language tone, with Instructor Similarity scores of approximately 0.92 across scenarios, underscoring the importance of prompt design in effective summarization tasks.

The findings of this research emphasize the critical role of prompt design in optimizing model efficacy and point to the significant potential for further exploration in prompt engineering. Moreover, the study advocates for the standardized integration of such advanced LLMs in healthcare practices, highlighting their potential to enhance the efficiency and accuracy of medical documentation.

## 1. Introduction

Large language models (LLMs) are designed to process and generate human-like text on a large scale [1]. These models, such as GPT-3.5 and GPT-4, are trained on massive amounts of various language data, enabling them to understand, interpret, and generate text across several domains [2]. LLMs are utilized to automatically generate concise impressions based on written radiology reports [3–9]. LLMs are valuable tools for improving efficiency in the understanding of medical findings due to their ability to find contextual meaning, understand medical terminology, and generate coherent impressions.

In the dynamic evolution of artificial intelligence (AI), generative models have appeared as promising tools, specifically in natural language processing (NLP). Generative LLMs, due to their ability to process multimodal inputs combining both images and text, present a promising advancement in the medical field [10]. This is vital as the medical field relies on various data types such as medical imaging for informed decision-making [10]. These models, such as GPT-3.5 and GPT-4, exhibit exceptional abilities such as zero-shot learning and few-shot learning, representing good performance even without training in medical data. In the medical imaging field, these models show versatile abilities, not only extracting radiological findings but also structuring reports and generating impressions based on the written findings or the images themselves [4, 9, 11, 12]. Although this study demonstrates significant potential for LLMs in assisting radiologists, their deployment in real-world clinical environments requires careful consideration of adaptability to various medical settings and the need for specialized training of clinical personnel. Healthcare settings vary in their workflows, data formats, and terminologies, meaning that customized implementations of these models may be necessary to ensure reliability and safety in clinical practice.

While generative LLMs have made considerable advancement in impression generation, several key challenges persist, notably the issues of data bias and inconsistent quality in open-source radiology datasets. While valuable, open-source datasets suffer from variations in reporting standards and quality, which can further hinder model reliability and accuracy when applied in clinical settings. Also, although LLMs like GPT-3.5 and GPT-4 have shown considerable promise in medical applications, inherent data biases and the inconsistent quality of open-source radiology datasets remain key challenges. These models are typically trained on vast, nondomain-specific datasets, which may not fully capture the nuances of clinical language or the variability in radiological reporting. These challenges can reduce the accuracy and generalizability of LLM outputs, making them less reliable in clinical settings.

**Figure 1:**
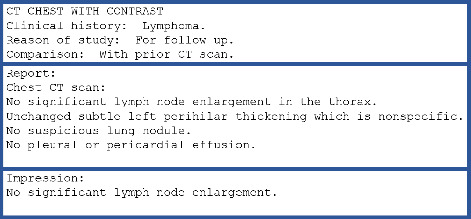
Schematic representation of a typical radiology report structure, illustrating the three key sections: background information detailing the examination and patient particulars, the findings section which provides a comprehensive description of the radiological observations, and the impression section which encapsulates the primary findings.

Our study tackles these issues by using high-quality, real-world radiology reports from the King Hussein Cancer Center (KHCC), which enhances the clinical relevance of the generated outputs. Furthermore, our research highlights the often-overlooked importance of prompt design in LLM performance, offering a systematic comparison of different prompt strategies, including zero-shot, few-shot, and retrieval-augmented generation (RAG) methodologies, to improve accuracy and contextual relevance in clinical applications. In the field of medical imaging, the transmission of information between radiologists and physicians relies on radiology reports. Radiology reports usually have three parts: the background section explaining the exam and patient details, the findings section which gives a detailed description of observations, and the impression section, which gives a summary of the main findings [13] (Figure 1). The impression is vital in guiding further treatments and making informed decisions [14]. However, manually writing these impressions can be time-intensive and prone to errors, particularly for less experienced radiologists [13]. This is why using automatic impression-generating systems can significantly improve the quality of clinical reporting.

## 2. Background and Related Work

The field of NLP has observed an extraordinary transformation with the advent of LLMs like GPT-3 [15], GPT-4 [16], and BERT [17]. These models play a vital role in improving language understanding, processing, analysis, and generation abilities [18].

The field of NLP has made substantial advancement through the integration of new technologies, particularly the fine-tuning of pretrained models. The BERT model, utilizing Transformer architecture, represents one of these important advancements [17, 19]. Preceding BERT was GPT-1, which marked an early advancement into LLMs with its 117 million parameters and training methodologies that combined self-supervised learning with supervised fine-tuning, leveraging transformer decoder architecture [20]. GPT-1 performed well in tasks like understanding natural language and answering questions. Then, BERT-Large, a larger version, that had 340 million parameters was introduced by Google which had high performance through innovations like Mask Language Modeling and Next Sentence Prediction [17].

Following the innovative BERT, the GPT-2 model was launched with a remarkable increase in scale to 1.5 billion parameters, setting a new benchmark in model complexity [21]. The GPT series continued to evolve with GPT-3, which grew to 175 billion parameters [20] offering a wide range of training abilities, from unsupervised learning to more targeted strategies like zero-shot and few-shot learning [15, 20]. As the GPT series progressed, GPT-4 developed as a commercial successor to GPT-3.5 with expanded capabilities.

The generative LLMs, including ChatGPT, based on GPT-3.5, and GPT-4, have demonstrated a profound ability to engage in human-like conversation and generate clear text. ChatGPT, in particular, benefits from the Reinforcement Learning from Human Feedback (RLHF) technique, which fine-tunes the model's output through iterative human input, enhancing its contextual understanding [22]. These advancements in generative LLMs, particularly with GPT-3.5 and GPT-4, have not only improved their learning efficiency through in-context examples but have also expanded their applicability across various domains, including education and healthcare, where they excel in tasks such as text classification and summarization [15, 23, 24].

### 2.1. Application of LLMs in the Healthcare Sector

Generative LLMs like GPT-3.5 and GPT-4 hold significant promise for enhancing medical applications, particularly by assisting healthcare professionals (HCPs) in creating detailed and accurate patient records. These records can include symptoms, medical notes, and results from laboratory tests and imaging [6], potentially reducing the load of manual documentation and improving healthcare efficiency [6].

Furthermore, generative LLMs can support medical decision-making by offering personalized drug recommendations and helping in the selection of appropriate diagnostic tests, thus enabling informed decision-making [11]. Another aspect where LLMs play a vital role in patient-centered care is by providing reliable medical information that can be retrieved from literature and guidelines [8, 9]. Also, LLMs can assist in health-related education and training for HCP patients and their caregivers [8].

There is a growing application for LLMs in the domain of radiology [3, 5, 9, 11–13, 18, 24–33], where models such as ChatGPT and GPT-4 are used for summarizing radiology reports and generating impressions. This task, a subset of abstractive summarization, helps extract the primary findings from reports, enabling physicians to make well-informed decisions [34].

Various model architectures, such as seq2seq models and BERT-based methods like BioBERT and Clinical BERT, showed exceptional accuracy in radiology report summarization and impression generation [18, 31, 32]. For instance, You et al. [35] used MIMIC-CXR [36] and OpenI [37] datasets to establish a focus-attention mechanism and saliency-selection network into the encoder and decoder for enhanced abstractive summarization. Li et al. proposed a method for keywords-guided abstractive sentence summarization, applying a dual-attention and dual-copy mechanism [38].

Generative LLMs were used extensively in impression generation, Jiang et al. introduced an iterative optimization algorithm for impression generation, achieving state-of-the-art performance on MIMIC-CXR and OpenI datasets without additional training or LLM fine-tuning [25]. Furthermore, experimental results of the ChatGPT-based Contrastive Learning (CCL) method on OpenI and MIMIC-CXR datasets establish the effectiveness of CCL in enhancing impression generation [26].

Despite the advancement of impression generation through generative LLMs has been notable, there is a critical issue that remains largely unaddressed: the prevalence of data bias and the variable quality within open-source datasets for radiology reports. Such limitations can impact the accuracy of the generated impressions [26]. To address this gap, our study uses radiology reports extracted from the electronic medical record of KHCC, ensuring a thorough evaluation of the reports' quality. The increasing popularity of generative LLMs highlights the need for a comprehensive evaluation of their performance in radiology report annotation and impression generation. This study is aimed at comparing the effectiveness of GPT-3.5 and GPT-4 in, generating accurate impressions using different prompts and approaches, including zero-shot, few-shot, and RAG methodologies.

## 3. Methodology

The temporal scope of patient data used in this study ranges from 2014 to 2023. This duration allows for a comprehensive source for data extraction and analysis of patient records. The data utilized in the study was collected from Electronic Health Records at the KHCC. All patient data used in this study were deidentified and handled following the ethical guidelines established by the KHCC's Institutional Review Board (IRB) (23 KHCC137). The data were stored on a password-protected computer, and only the research team had access to this information, ensuring that patient privacy was strictly maintained throughout the study. The utilized dataset includes the findings along with the corresponding impression of radiological computed tomography (CT) Chest scans. The impression section provides a concise summary and interpretation of the findings usually written by the attending radiologist. The study included a sample size of 100 deidentified CT Chest Scan reports. To ensure the quality of data, reports were filtered by the names of senior radiologists that contribute to a higher quality of interpretation and impression. To ensure adherence to privacy and ethical considerations, all identifiers within the reports were anonymized and encrypted.

For data preprocessing, regex methods were used to separate the impression from findings generating two separate texts for each patient. The findings were used as input inserted within the prompt, where the impressions were used as ground truth and reference against the outputs of our model.

The main methodology of this work is characterized by the utilization of open AI GPT models through API access. Previous research has rigorously assessed the baseline medical knowledge of both GPT-3.5 and GPT-4, demonstrating clear advancements in GPT-4. For instance, a study by Microsoft Research evaluated these models on the United States Medical Licensing Examination (USMLE), revealing that GPT-4 not only exceeded the passing score by over 20 points but also outperformed specialized models fine-tuned for medical knowledge. This indicates that GPT-4 possesses a significantly stronger foundation in medical reasoning and content accuracy compared to GPT-3.5 [39]. Additionally, another study comparing their performance on the German Medical Licensing Examination showed that GPT-4 achieved an average score of 85%, ranking within the top percentiles among medical students, whereas GPT-3.5 demonstrated inconsistent and often inadequate performance [40]. These findings confirm that GPT-3.5 and GPT-4 have demonstrated pre-existing medical knowledge, which supports its potential ability to generate accurate medical impressions. Therefore, our study builds on this established evidence, focusing on the comparative performance of GPT-3.5 and GPT-4 in medical impression generation without conducting a redundant pre-existing knowledge test.

The study's methodology consists of two distinct techniques. The first technique involves in-context learning (ICL), which is a method of directing LLMs on novel tasks through prompting in a natural language format.

Generally, a common method of tailoring LLMs to a certain domain is training an existing model with a training dataset that is employed to update the model's parameters through a backpropagation algorithm. However, there have been several research and investigations on the efficacy of taking advantage of ICL instead, which proves to be advantageous for several reasons. ICL allows the model to address new LLM tasks without fine-tuning, thereby, requiring less computational resources, time, and effort.

In the second technique, the RAG method is employed. In such a method, findings are queried using predefined questions set by an expert radiologist. The response to each query is then resent into open-AI LLM with the instruction of summarizing them into concise sentences. The main goal of applying these two methods is to evaluate several techniques that may prove to be just as efficient, if not more, than fine-tuning LLMs, exploring alternatives to reduce the need for extensive computation resources.

### 3.1. In Context Learning Prompt Generation

**Figure 2:**
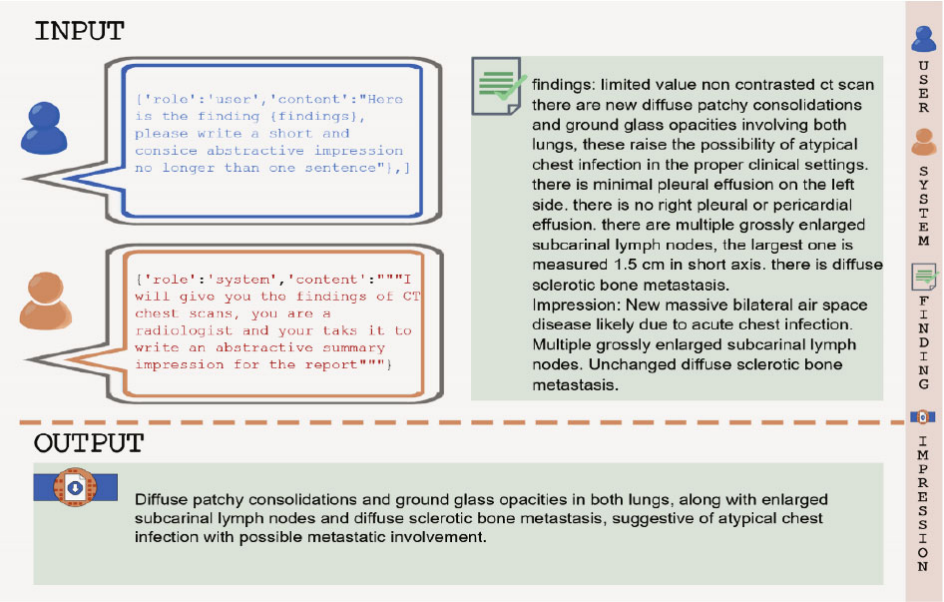
An example of autonomous impression generation in a zero-shot scenario using Prompt 1.

**Figure 3:**
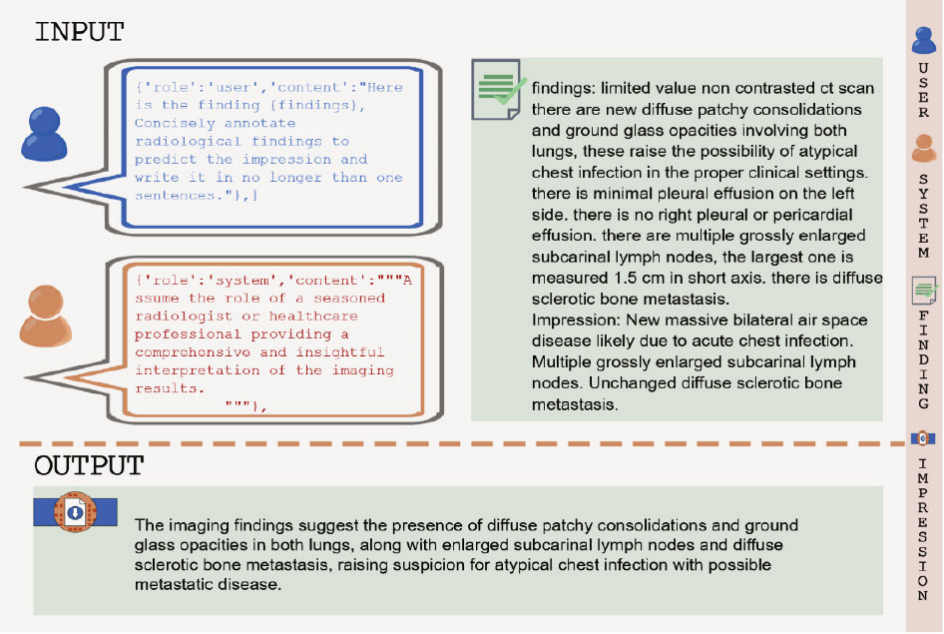
An example of autonomous impression generation in a zero-shot scenario using Prompt 2.

**Figure 4:**
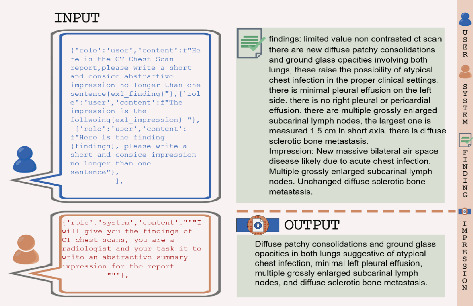
An example of autonomous impression generation in a few-shot scenario using Prompt 1.

**Figure 5:**
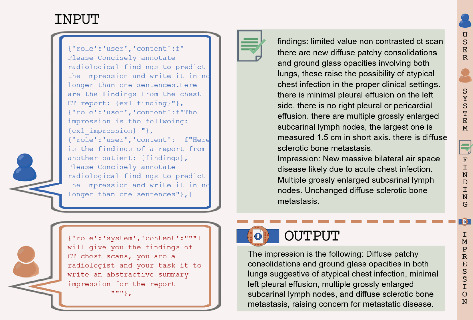
An example of autonomous impression generation in a few-shot scenario using Prompt 2.

LLMs are often described as “black boxes” due to the complexity of their underlying architecture, which makes it challenging to interpret how specific inputs (prompts) influence their outputs. To address this challenge, prompt optimization is employed, which involves designing, refining, and testing various prompts to observe and improve how they impact the model's output. This process helps bridge the gap between human input and the model's responses, allowing for more controlled and effective use of these powerful tools. There are two primary techniques for prompting: zero-shot and few-shot. In the zero-shot approach, the model is given a direct task request without any examples. For instance, the model might be asked to generate an impression based solely on a patient's radiological findings, relying entirely on its pre-existing knowledge. In contrast, the few-shot approach involves providing the model with one or more examples of finding-impression pairs within the prompt. These examples serve as a reference, guiding the model to produce more accurate and contextually relevant outputs. In this study, we applied the few-shot approach by presenting the GPT models with one exemplary sample of finding-impression pairs to guide their output. In our study, we utilized two primary prompts, Prompt 1 and Prompt 2, each applied in both zero-shot and few-shot scenarios. Prompt 1 is designed to produce a concise, one-sentence summary of the radiological findings, asking the model to “generate a short and concise abstractive impression.” This prompt is direct and focused on briefness. Prompt 2, on the other hand, instructs the model to ‘assume the role of a seasoned radiologist or HCP' and provide a comprehensive and insightful interpretation of the imaging results.' This prompt encourages a more detailed and thoughtful response, drawing on the model's broader knowledge base. As a demonstration of these techniques, Figures 2, 3, 4, and 5 present visual examples of prompts along with their corresponding outputs, serving to explain the concept of prompt engineering.

### 3.2. RAG

**Figure 6:**
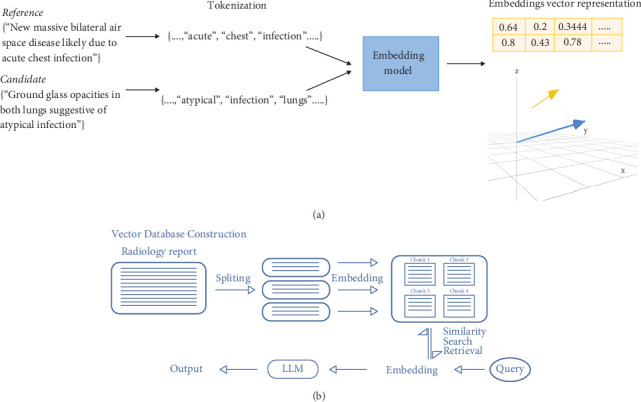
Retrieval-augmented generation (RAG) pipeline. (a) Conversion of radiological report text into a machine-readable vector format. (b) Process of creating a vector database from radiology reports.

The LLM's knowledge base is dependent upon the data that was used for training, or as discussed before, its knowledge base can be slightly modified using ICL. The main objective behind RAG is to provide an external knowledge base that can interact with external datasets. This method consolidates the LLM's response to domain-specific queries. A pipeline that queries through stored patients' radiological findings to write the final impression was proposed. The pipeline, as shown in Figures 6(a) and 6(b), consists of three main tools: a database vector, LangChain [41], and openAI GPT-3.5-turbo model. In our study, we implemented RAG specifically with the GPT-3.5 model only.

### 3.3. Retrieval Pipeline

#### 3.3.1. Note Splitting

The utilization of a vector database overcomes the 4096 token limit context window of GPT-3.5-turbo, by splitting the notes into multiple chunks. In our dataset, however, the average token length of the reports is 131 which does not exceed the limit. But to prevent any errors, the notes were split based on the number of characters. Every 100 characters in the note were split with a 20-character overlap between them to ensure the continuity of the context. Text splitting was done using LangChain python package Version 0.0.352, which is a framework for developing applications powered by language models.

#### 3.3.2. Chunk Embedding and Storage in a Vector Database

The provided chunks are then embedded using the Instructor Embeddings Model developed by the University of Hong Kong which has shown that it generates task-specific embeddings that achieve state-of-the-art performance. Embeddings are the numerical representation of text in high-dimensional vectors in a continual vector space. Embeddings capture the semantic meaning of text and its relation to other texts. This allows vector space to understand the context of data and aids in semantic similarity searches. Subsequently, the embeddings are then stored in the vector database Pinecone. Pinecone is a database that stores these high-dimensional vectors, ensuring efficient database management and ease of subsequent retrieval tasks. In this approach, the vector database serves a dual purpose in the framework. Firstly, it provides a structured repository for efficient storage and retrieval of information, supporting subsequent stages of the framework. Secondly, the vector database plays a crucial role in optimizing the performance of the language model as it simulates a “long-term memory” and helps address the token limits in LLMs.

#### 3.3.3. Information Retrieval and Extraction

Each report is funneled through a predefined set of questions that ultimately determine the main elements that need to be addressed in the impression section of radiological reports. The questions were set by an expert radiologist and reviewed to ensure their comprehensiveness. The questions are queried against the vector space with the stored embeddings to extract relevant information. The LangChain package served as a useful tool to connect the vector store with the LLM. The package embeds the query and initiates a similarity search to conduct textual parallels to existing embedded text in the vector store. The similarity search is set to the top 5 text chunks that are in the highest similarity to the requested information. The query and the retrieved chunks are subsequently converted back to text form and fed into the GPT OpenAI, which responds to the query. This approach ensures that only the query and the relevant chunks of information contribute to the dialogue prompt.

#### 3.3.4. N-Gram-Based Metric

Recall-oriented understudy for gisting evaluation (ROUGE) is a commonly used metric to evaluate summarization comparing the reference, provided as the ground truth with the candidate, generated by the language model. ROUGE is an N-gram-based model that calculates the overlap of words and sentences between the generated text and the reference text. It is subdivided into three different scores: ROUGE-1, ROUGE-2, and ROUGE-L. Unigram overlap (single words) between two texts results in ROUGE-1, while ROUGE-2 is a bigram measurement that calculates the overlap of pairs of consecutive words. At the same time, ROUGE-L looks for the longest common sequence in alignment with the reference sentence. The combination of three scores allows for a comprehensive overlook of text similarity. Nevertheless, this is a very surface-level comparison of texts especially in cases where the generated summary is abstractive. ROUGE metric looks for similar words and subphrases within two texts and may not adequately account for variations in word order. In many cases, two words may not be exact matches but are synonyms of one another, thereby ROUGE cannot distinguish the overlap without considering their contextual relationships. This calls for the utilization of a model that captures the semantic meaning of text that is less sensitive to word order and variation.

#### 3.3.5. Semantic Embedding-Based Metrics

To overcome the limitations of ROUGE as a metric for evaluation, a commonly used method is to evaluate text similarity using text embedding. BERTScore which is a language generation metric that is trained on BERT contextual embedding. Text is tokenized where sentences are broken down to words or even subwords. BERT uses a technique called WordPiece tokenization, which allows the model to handle out-of-vocabulary words. Then, the embedding of each token is obtained, where they are represented as vectors. Then cosine similarity is applied within vectors to assess the similarity between two pieces of text. The prominent advantage of contextual embedding is the ability of the models to represent the same tokens with different vectors depending upon their position and meaning within a sentence. This effectively tackles the drawbacks of n-gram-based metrics that look for exact matches of string and miss out on capturing word synonyms and semantic similarities. Also, the instructor similarity-trained model was deployed to obtain similarity metrics. What sets this model apart is its task and domain-specific dataset that it has been trained on. Each input of the Instructor training dataset is coupled with a specifically defined task. In other words, not only does this model generate different embedding based on semantic meaning, but also can generate different embedding based on the given task, thereby emphasizing its flexibility.

## 4. Results

Our study evaluates the performance of ICL of GPT-3.5, GPT-4, and the RAG methodology in the context of radiology findings summarization and impression generation. Our evaluation technique includes multistep comparison using various metrics including ROUGE-1, ROUGE-2, ROUGE-L, BERTScore, and Instructor Similarity. The effect of using different prompts and approaches (zero-shot and few-shot) on each model's report summarization and impression-generation abilities was explored.

### 4.1. Descriptive Analysis

Table 1 presents a comprehensive evaluation of GPT-3.5, GPT-4, and RAG methodologies in processing radiology reports. The evaluation metrics include ROUGE-1, ROUGE-2, ROUGE-L, BERTScore, and Instructor Similarity, reflecting various aspects of language model performance from syntactic coherence to semantic alignment and domain-specific accuracy.

In the few-shot scenario using Prompt 2, GPT-3.5 achieves the highest average performance across the ROUGE metrics. Specifically, the average ROUGE-1 score is 0.41 with a standard deviation of 0.13, suggesting consistency in capturing the exact wording from the source text. The ROUGE-2 score averages 0.22, and the ROUGE-L at 0.32, both with a standard deviation of 0.11, indicating a strong ability to reflect the same sentence structure as the reference texts.

Conversely, the RAG methodology outperforms other models in the BERTScore metric, achieving an average score of 0.92 with a minimal standard deviation of 0.02, which implies a high degree of semantic similarity between the generated impressions and the reference standard. When it comes to Instructor Similarity, RAG shows an impressive average score of 0.90, matching closely with the expert radiologist's perspective, and this is consistent as evidenced by the low standard deviation of 0.02.

In the zero-shot scenario, GPT-4 shows strong performance with Prompt 1, averaging 0.35 on ROUGE-1 with a standard deviation of 0.17, suggesting moderate consistency. However, it is in the Few-shot scenario with Prompt 2 where GPT-4 shines, with a ROUGE-1 average of 0.30, ROUGE-2 of 0.16, and ROUGE-L of 0.23, all with a standard deviation of 0.13 or less, indicating reliable performance across multiple evaluations.

Table 1 suggests that while GPT-3.5 excels with specific prompting in few-shot scenarios, RAG consistently provides high semantic accuracy. GPT-4's performance is stable across different prompts and scenarios, but it does not reach the semantic accuracy levels of RAG, particularly in the BERTScore and Instructor Similarity metrics.

These results highlight the importance of prompt selection in Few-shot learning for optimizing model performance. Moreover, they underscore the RAG method's robustness in producing semantically rich and domain-accurate outputs, potentially due to its ability to draw from an external knowledge base.

After the descriptive analysis was conducted, the second stage of analysis was performed. This stage included a series of statistical analyses aimed at evaluating the performance of each model under several scenarios, which combined the use of two different prompts and two methodologies: zero-shot and few-shot. To assess performance variation within the models, the Lilliefors test was applied to determine normality, which was then followed by either paired t-tests or Wilcoxon tests, depending upon the results of the normality assessment.

### 4.2. GPT-3.5

#### 4.2.1. Evaluation of the Impact of Using Different Prompts

The data presented in Table 2 highlight the comparative effectiveness of different prompts used in the zero-shot and few-shot learning approaches. In the zero-shot context, Prompt 1 demonstrated a statistically significant advantage in ROUGE-1, ROUGE-L, and Instructor Similarity with *p* values of 0.0237, 0.0001, and 0.0297, respectively. These metrics suggest that without prior examples, Prompt 1 is particularly effective in producing responses from the language model that are aligned with human-generated summaries.

Furthermore, in the few-shot approach, the dominance of Prompt 1 persisted uniquely in the BERTScore metric, as indicated by a *p*-value of p ≤0.001, reported in Table 2. This implies that when provided with examples, Prompt 1 leads to semantically richer outputs as compared to Prompt 2, closely reflecting the reference standards used for evaluation.

#### 4.2.2. Evaluation of the Impact of Using Different Approaches

The analysis shows that using Prompt 1 in both approaches did not yield significant differences in the model's performance, as indicated by the *p* values across all metrics, in which *p* values were not below 0.05 which would suggest statistical significance. On the other hand, using Prompt 2 in the few-shot approach resulted in statistically significant performance improvements. This is shown by lower p-values in ROUGE-1 (*p* = 0.0009), ROUGE-L (*p* = 0.0003), and BERTScore (*p* ≤ 0.001). Furthermore, instructor similarity also had a significant increase (*p* = 0.0140), indicating a greater alignment with expert evaluations. These results, detailed in Table 3, underscore the impact of prompt design on the effectiveness of GPT-3.5, GPT-4, and RAG methodologies in impression generation tasks in different approaches.

### 4.3. GPT-4

#### 4.3.1. Evaluation of the Impact of Using Different Prompts

In Table 4, the statistical analysis outlines a clear advantage of using Prompt 1 with GPT-4 in the few-shot approach, where it significantly outperforms across all metrics. This is evidenced by the *p*-values, which are exceptionally low (*p* ≤ 0.05) for all ROUGE metrics and *p* ≤ 0.001 for BERTScore), indicating a strong statistical significance. Such results suggest that Prompt 1 is highly effective in guiding GPT-4 to generate outputs that are both syntactically and semantically aligned with the expected standards.

Furthermore, in the zero-shot approach, Prompt 1 continues to demonstrate remarkable performance, particularly in capturing the structure of the reference text as shown by ROUGE-L (*p* = 0.0005) and in aligning with expert opinion in terms of Instructor Similarity (*p* = 0.0297). These findings suggest that Prompt 1, even without the benefit of example inputs, can direct the GPT-4 model to produce highly accurate summaries and impressions.

The results highlight the critical role that prompt design plays in the effectiveness of language model outputs, especially when minimal context is provided. The significant difference in performance between the few-shot and zero-shot approaches for Prompt 1 also indicates the potential of few-shot learning to enhance the model's output, making it an essential tool for tasks requiring high levels of linguistic precision and domain expertise.

#### 4.3.2. Evaluation of the Impact of Using Different Approaches

Table 5 provides a statistical evaluation of the impact of using different approaches with Prompts 1 and 2 on the language model's performance. Consistent with the results observed in GPT-3.5, the utilization of Prompt 1 in GPT-4 did not yield significant differences in performance metrics, as indicated by the higher *p*-values in ROUGE-1 and BERTScore. In contrast, the use of Prompt 2 discovered a notable variation in performance. Specifically, in the few-shot approach, a significant improvement was seen in instructor similarity with a *p* value of 0.014. Furthermore, the zero-shot approach with Prompt 2 demonstrated superior performance in ROUGE-1 (*p* = 0.0131) and ROUGE-L (*p* = 0.0446), suggesting a strong ability to match the reference text in both fine-grained detail and overall structure. Notably, BERTScore achieved a p-value of 0 with Prompt 2 in the zero-shot approach, indicating a perfect semantic alignment with the reference. These results, summarized in Table 5, highlight the influence of prompt design on the efficacy of language models in generating text that is consistent with human standards and expert evaluations.

### 4.4. Comparison of GPT-3.5 (With RAG), GPT-3.5, and GPT-4

Prompt 1 outperformed Prompt 2 in the majority of scenarios, we focused on Prompt 1 in our subsequent comparison. In this step, we compared the performance of GPT-3.5, GPT-4, and the RAG method in both the zero-shot and few-shot approaches. We performed a one-way ANOVA test to evaluate variability. Afterward, given a significant difference, post hoc comparisons were conducted using the Tukey test.

#### 4.4.1. Zero-Shot Approach

**Figure 7:**
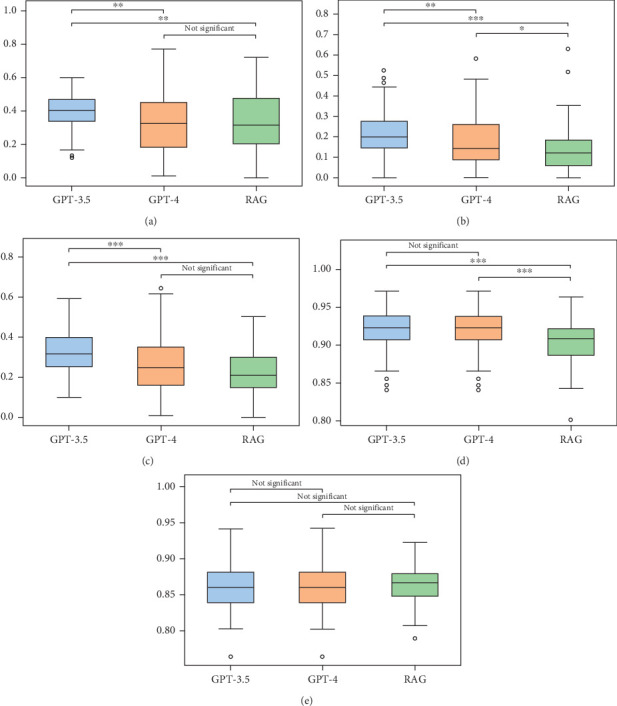
Comparison of GPT-3.5, GPT-4, and RAG method in zero-shot learning: a box plot analysis. (a) ROUGE-1 score, (b) ROUGE-2 score, (c) ROUGE-L score, (d) Instructor Similarity score, and (e) BertScore.

The statistical analysis shows that there are significant differences in performance across most metrics among the models. As shown in Figure 7(a), GPT-3.5 generally shows superior performance in ROUGE-1, outperforming GPT-4 and RAG with mean differences of 0.0652 and 0.0607, respectively, and demonstrating statistically significant results (*p* = 0.0136 for GPT-4 and *p* = 0.0237 for RAG). Similarly, Figure 7(b) shows that GPT-3.5 leads in ROUGE-2 with a mean difference of 0.071 over RAG (*p* < = 0.001). ROUGE-L scores are highest for GPT-3.5, followed by GPT-4, and lastly RAG, with mean differences of 0.0583 between GPT-3.5 and GPT-4 (*p* = 0.0037), and a noticeable difference between GPT-4 and RAG (*p* < = 0.001) as prescribed in Figure 7(c). The analysis further shows a significant difference between GPT-3.5 and RAG (*p* = 0.0511) in ROUGE-L.

In the assessment of Instructor Similarity shown in Figure 7(d), there is no notable difference between GPT-3.5 and GPT-4 (*p* = 1.0), suggesting comparable performance between these models in aligning with expert evaluations. However, both GPT-3.5 and GPT-4 show a statistically significant advantage over RAG in this metric (*p* = 0.0001).

The boxplots visualize the central tendency and dispersion of scores, providing a visual confirmation of the statistical findings. Notably, BERTScore does not exhibit significant differences among the models, suggesting a uniform performance in semantic similarity across GPT-3.5, GPT-4, and RAG in the zero-shot approach (Figure 7(e)). These results, summarized in Figures 7(a), 7(b), 7(c), 7(d), and 7(e) highlight the nuanced abilities of each model in the context of text generation without prior examples.

#### 4.4.2. Few-Shot Approach

**Figure 8:**
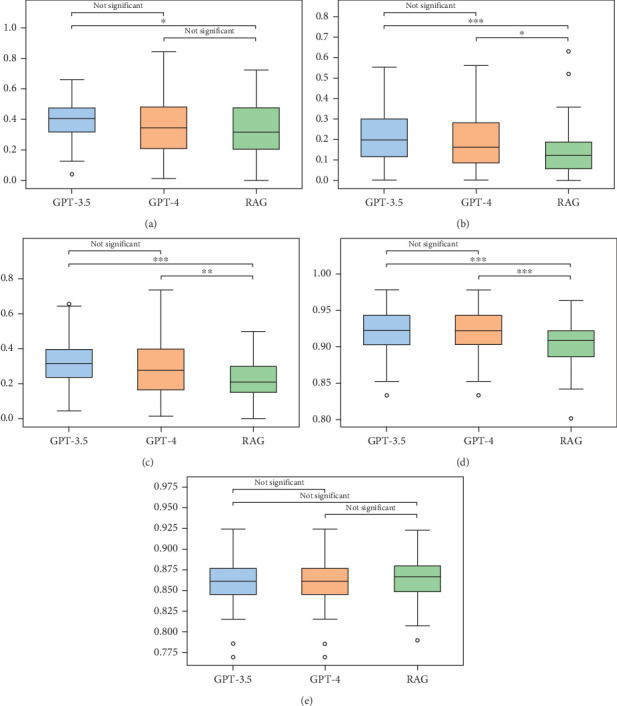
Comparison of GPT-3.5, GPT-4, and RAG method in few-shot learning: a box plot analysis. (a) ROUGE-1 score, (b) ROUGE-2 score, (c) ROUGE-L score, (d) Instructor Similarity score, and (e) BertScore.

Figures 8(a), 8(b), 8(c), 8(d), and 8(e) present a visual comparison of the performance distributions for GPT-3.5, GPT-4, and RAG in a few-shot scenario across multiple evaluation metrics: Figure 8(a) for ROUGE-1, Figure 8(b) for ROUGE-2, Figure 8(c) for ROUGE-L, Figure 8(d)) for Instructor Similarity and Figure 8(e) for BERT Score. Significant differences are noted particularly in the ROUGE-1 and ROUGE-L metrics, where GPT-3.5 (*p* = 0.0007) and GPT-4 (*p* = 0.0228) have statistically outperformed RAG, indicating their higher capability to capture key phrases and sentence structures as compared to RAG. The lack of significant difference in ROUGE-2 between GPT-3.5 and GPT-4 (*p* = 0.529) suggests similar performance levels in capturing bigram overlaps. Additionally, the superior performance of both GPT-3.5 (p ≤ 0.001) and GPT-4 (*p* = 0.0055) over RAG in the ROUGE-L metric highlights their effectiveness in generating long-form content that exactly matches the reference data.

In terms of Instructor Similarity, both GPT-3.5 and GPT-4 again outperform RAG significantly (*p* = 0.0002), which implies that their generated content aligns more closely with expert evaluations. The equivalent performance between GPT-3.5 and GPT-4 (*p* = 1.0) in Instructor Similarity suggests that they are comparably capable of reflecting expert opinion in their outputs.

## 5. Discussion

In our study, the ICL abilities of GPT-3.5, GPT-4, and RAG in summarizing radiology reports and generating impressions were examined. In the NLP landscape and LLMs, this task is critical, as the accuracy of the generated impressions directly impacts patient care. Generated impressions were evaluated using ROUGE metrics, BERTScore, and Instructor Similarity.

Based on our results, it is obvious that GPT-3.5 and GPT-4 models along with the RAG method show different performance characteristics. The ROUGE-1, ROUGE-2, and ROUGE-L metrics, which measure the overlap of unigrams, bigrams, and the longest common subsequence between the generated and original impressions, show that GPT-3.5 models tend to perform slightly better in the few-shot approach compared to zero-shot, specifically in the ROUGE-1 metric, suggesting a higher unigram overlap. This is consistent with the hypothesis that few-shot learning provides more context to the model, thus improving its ability to generate summaries that are closer to the reference texts [42]. Moreover, the Instructor Similarity, which measures the alignment of generated impressions with expert expectations based on the semantic meaning, exhibits high mean scores for GPT-3.5, GPT-4, and RAG methods across both prompts, with a low standard deviation. This consistency highlights the models' strength in catching the semantic meaning of the task as proposed by experts [43]. Furthermore, the BERTScore results, which capture semantic similarity via contextual embeddings, were moderately high for GPT-3.5 and GPT-4, which proposes that the generated impression is semantically aligned with the original impression [44]. Interestingly, the RAG method establishes a distinguished performance with the highest BERTScore, demonstrating its effectiveness in semantic matching, likely due to its retrieval-augmented mechanism [44]. Particularly, the RAG method not only outperformed in BERTScore but also achieved a high Instructor Similarity score, indicating its ability to generate contextually relevant impressions despite the lower n-gram overlap that was reflected by moderate performance in the ROUGE metrics [44].

GPT-3.5 and GPT-4 exhibited comparable performance in the zero-shot approach, but both showed significantly better performance over RAG in ROUGE-1 and Instructor Similarity. This proposes that even though RAG is semantically strong, GPT-3.5 and GPT-4 may be better at detecting the tone of language that is vital for summarizing complex medical reports, without needing training or fine-tuning.

In contrast to the zero-shot approach, the performance of GPT-3.5 and GPT-4 was relatively similar in the few-shot approach (*p* = 0.529). This indicates that when the model is provided with examples, the model will generate impressions with relatively similar efficacy and accuracy [15, 26]. While the GPT-4 model is more advanced, there is a limit to excelling at summarization tasks. No significant difference will be detected between either model as there exists a threshold beyond which larger parameters of a model can contribute to the overall efficacy. Optimization within prompts and ICL may prove to have a larger impact on summarization tasks rather than model size to achieve advancements.

GPT-4's performance exhibited variability; it outperformed prompt 1 in few-shot scenarios across all metrics, supporting the ability of few-shot learning to improve model output, as previously proved by Brown et al. [15]. On the other hand, GPT-4's performance with Prompt 2 in the zero-shot scenario was distinguished, specifically in ROUGE-1 and ROUGE-L. This observation suggests that robust prompts may play a crucial role in the model's efficacy enhancement in radiology report summarization and generating impressions, even in the absence of any ICL. Nevertheless, there still needs to be more extensive methods of analyzing LLM outputs based on engineered prompts. There have been several methods proposed by previous literature work, such as error analysis and manual evaluation [45].

Interestingly, during our prompt testing process, as radiological reports were fed into LLMs, there was an apparent exhibition of hallucinations. One example was where the impression (output) had a mention of an X-ray of a fractured risk. Upon evaluation, this was nowhere to be found within the reports. The main reason behind such outputs was found to be due to an error within the RAG pipeline where the portion of the radiology reports was delayed and incoming after the queries. While this was due to a technical error, it still provides insight into the lack of accuracy of LLMs in situations of insufficient information. It has been shown that one of the main methods to eliminate hallucinations is to provide the LLM with enough information so that it is not triggered to fabricate it. Researchers demonstrated that to mitigate hallucinations, it is prudent to underline the effect of information availability that grounds the LLM with contextual input and understanding minimizing inaccuracies and increasing overall reliability.

Our study has various impacts on the application of LLMs in the medical field. The ability of these models to generate accurate and precise impressions with minimal or without training proposes an efficient way for impression generation which can reduce the radiologist workload and improve the timeliness of patient care workflows. Moreover, the high Instructor Similarity scores across models indicate the model's reliability in clinical work.

Our study explored multiple prompt engineering techniques, including zero-shot, few-shot, and RAG approaches, to evaluate the impact of different prompt designs on the performance of GPT-3.5 and GPT-4. Zero-shot prompts required the models to generate impressions without prior examples, while few-shot prompts provided one or more example pairs to guide the output. The RAG method was employed to integrate domain-specific knowledge from external sources. These approaches demonstrated that the structure and specificity of prompts significantly affect model performance. However, prompt sensitivity remains a key challenge, particularly when dealing with different report formats and clinical contexts. The study also shows the importance of prompt design and the potential need for domain-specific fine-tuning, as shown by the differences in performances across different prompts and approaches. This is vital in the medical field, where the cost of misinterpretation is very high. Despite the promising results demonstrated by GPT-3.5, GPT-4, and the RAG method in generating radiology report impressions, several limitations must be acknowledged in this study. First, the data used were sourced exclusively from the KHCC, which may limit the generalizability of our findings to other healthcare settings. While the dataset reflects real-world clinical practice, it is drawn from a specific geographical and institutional context that may not fully represent the diversity of patient populations or imaging protocols in other regions. This poses a challenge when attempting to apply these models universally across different institutions, demographics, or imaging modalities. Furthermore, although the performance of the models was impressive based on automated metrics such as ROUGE and BERTScore, these metrics do not fully capture the clinical relevance of the outputs. LLMs, by their nature, can occasionally generate incorrect or fabricated information (“hallucinations”) and are sensitive to prompt variations, which could affect reliability in clinical environments. A more comprehensive evaluation, involving manual reviews by experienced radiologists, would be necessary to validate the clinical applicability and safety of these models. Future research should focus on expanding datasets to include data from multiple sources and geographic regions, refining prompt engineering techniques, and incorporating manual evaluations to further enhance the models' practical utility in clinical practice.

In addition, the practical application of LLMs in clinical settings presents challenges beyond data bias. Healthcare institutions employ different workflows, report formats, and terminologies, which may impact the adaptability of LLMs like GPT-3.5 and GPT-4. These models would likely require customization to align with the unique needs of various clinical environments. Additionally, integrating LLMs into clinical workflows will necessitate training HCPs in the use and verification of AI-generated reports. Ensuring proper training will be essential to enable the safe and effective use of these tools, particularly in high-stakes clinical decision-making. The high accuracy of LLMs, achieved through specific prompt designs, further raises concerns about their robustness. Our study demonstrates that prompt engineering can significantly improve model outputs, but this reliance on specific prompts suggests the model's performance could vary across different report formats and clinical contexts. Future research should explore the adaptability of these models across a broader range of radiology report structures, testing their robustness and consistency in real-world applications. Expanding the range of prompt testing and employing robustness verification techniques will be critical in determining whether these models can perform reliably in diverse medical environments.

Another important consideration is the ethical implications of using LLMs in clinical report generation, particularly regarding patient privacy and data security. Although this study employed de-identified and securely stored data, there remains a risk of unintentionally exposing sensitive information, especially when LLMs are deployed in clinical environments. The automated nature of these models also raises concerns about patient consent, data ownership, and the potential misuse of generated reports. Future research should prioritize the development of stricter guidelines and best practices to ensure the responsible use of LLMs in healthcare. Collaboration with legal and regulatory experts will be essential to ensure compliance with data protection laws such as HIPAA and to uphold the highest standards of patient confidentiality and security.

Finally, future research should focus on developing more adaptive prompt strategies to improve consistency across various clinical contexts. Refining few-shot and RAG methodologies, exploring automated prompt generation, and testing on larger, more diverse datasets will be crucial for improving robustness and scalability. Collaboration across institutions and regions, along with standardized training protocols, will be key to reducing bias and ensuring consistent model performance in different healthcare settings.

## 6. Conclusion

Our study contributes to the growing evidence supporting the potential of generative LLMs in the medical field. While the performance of these models is promising, especially in terms of syntactic and semantic alignment with reference texts, it is important to approach their use with caution due to the complexity of medical language and the critical nature of impression-generation tasks. The high Instructor Similarity scores suggest that these models can produce outputs closely aligned with expert expectations; however, this does not yet equate to full clinical reliability. Future work should focus on the integration of these models into clinical workflows, the development of prompts tailored to specific medical subdomains, and rigorous evaluation of their impact on clinical outcomes. Additionally, the training of open-source LLMs that rank highly on language benchmarks could be explored, as these models can be hosted locally, minimizing concerns about patient privacy and confidentiality.

## Figures and Tables

**Table 1:** Comparative descriptive analysis—Quantitative assessment of GPT-3.5, GPT-4, and retrieval-augmented generation (RAG) across zero-shot and few-shot learning scenarios using a variety of evaluation metrics. The metrics utilized for this analysis include ROUGE-1, ROUGE-2, ROUGE-L, BERTScore, and Instructor Similarity.

**Evaluation metrics**	**GPT-3.5**								**GPT-4**								**RAG**	
	**Zero-shot**				**Few-shot**				**Zero-shot**				**Few-shot**					
	**Prompt 1**		**Prompt 2**		**Prompt 1**		**Prompt 2**		**Prompt 1**		**Prompt 2**		**Prompt 1**		**Prompt2**			
	***μ***	**S** **t** **d**.	**μ**	**Std.**	***μ***	**Std.**	***μ***	**Std.**	***μ***	**Std.**	***μ***	**Std.**	***μ***	**Std.**	***μ***	**Std.**	***μ***	**Std.**
ROUGE 1	0.4	0.1068	0.3784	0.1308	0.3985	0.1266	0.4121	0.1233	0.3355	0.175	0.3183	0.1795	0.3593	0.1846	0.3031	0.1745	0.339	0.1779
ROUGE 2	0.2072	0.1157	0.1905	0.1126	0.2050	0.1295	0.2206	0.123	0.1699	0.1187	0.1563	0.1143	0.1852	0.1348	0.161	0.116	0.1362	0.1117
ROUGE L	0.3264	0.1077	0.2892	0.1171	0.3217	0.1261	0.327	0.1113	0.2681	0.1465	0.2383	0.1372	0.2871	0.1598	0.2352	0.1346	0.2259	0.1092
BERTS core	0.8597	0.0287	0.8614	0.0276	0.8606	0.0263	0.8497	0.0265	0.8597	0.0287	0.8614	0.0276	0.8606	0.0263	0.8497	0.0265	0.8648	0.0246
Instructor Similarity	0.9204	0.0263	0.9165	0.0286	0.921	0.0294	0.9225	0.9225	0.9204	0.0263	0.9165	0.0286	0.921	0.0294	0.9225	0.0268	0.9037	0.0287

**Table 2:** Evaluation of the impact of using different prompts—This table summarizes the statistical analysis conducted to assess the effects of using zero-shot and few-shot prompting techniques on the performance of language models.

**Metric**	**Approach**	**Test type**	**Lilliefors ** **p** ** value**	**p** ** value**
ROUGE-1	Zero	*t*-paired test	0.0606	0.0237
ROUGE-1	Few	Wilcoxon-signed-rank test	0.0415	0.3918
ROUGE-2	Zero	*t*-paired test	0.3055	0.0623
ROUGE-2	Few	Wilcoxon signed-rank test	0.001	0.1927
ROUGE-L	Zero	Wilcoxon signed-rank test	0.0035	0.0001
ROUGE-L	Few	Wilcoxon signed-rank test	0.0482	0.9587
Instructor Similarity	Zero	*t*-paired test	0.1182	0.0297
Instructor Similarity	Few	Wilcoxon signed-rank test	0.001	0.9313
BERTScore	Zero	Wilcoxon signed-rank test	0.0393	0.2244
BERTScore	Few	Wilcoxon signed-rank test	0.0026	≤ 0.001

**Table 3:** The results from a statistical analysis designed to understand the impact of using different approaches zero-shot and few-shot. The metrics used for evaluation are ROUGE-1, ROUGE-2, and ROUGE-L, which assess the overlap of the generated text with a reference, and BERTScore and Instructor Similarity, which measure semantic similarity and alignment with expert opinion, respectively.

**Metric**	**Prompt distribution type**	**Test type**	**Lilliefors *p*-value**	**p** ** value**
ROUGE-1	Prompt 1	*t*-paired test	0.7833	0.7903
ROUGE-1	Prompt 2	*t*-paired test	0.0789	0.0009
ROUGE-2	Prompt 1	Wilcoxon signed-rank test	0.025	0.9742
ROUGE-2	Prompt 2	Wilcoxon signed-rank test	0.001	0.0019
ROUGE-L	Prompt 1	*t*-paired test	0.2382	0.6259
ROUGE-L	Prompt 2	Wilcoxon signed-rank test	0.0313	0.0003
Instructor Similarity	Prompt 1	Wilcoxon signed-rank test	0.0262	0.5411
Instructor Similarity	Prompt 2	Wilcoxon signed-rank test	0.001	0.014
BERTScore	Prompt 1	*t*-paired test	0.8653	0.6845
BERTScore	Prompt 2	*t*-paired test	0.2054	≤0.001

**Table 4:** Evaluation of the impact of using different prompts—This table documents the outcomes of a statistical analysis assessing the efficacy of different prompts when used with the GPT-4 model in both few-shot and zero-shot approaches.

**Metric**	**Approach**	**Test type**	**Lilliefors *p*-value**	**p** ** value**
ROUGE-1	Zero	*t*-paired test	0.1662	0.0675
ROUGE-1	Few	*t*-paired test	0.5012	≤ 0.001
ROUGE-2	Zero	*t*-paired test	0.7408	0.0637
ROUGE-2	Few	*t*-paired test	0.38	0.0016
ROUGE-L	Zero	*t*-paired test	0.2253	0.0005
ROUGE-L	Few	Wilcoxon signed-rank test	0.0179	≤ 0.001
Instructor Similarity	Zero	*t*-paired test	0.1182	0.0297
Instructor Similarity	Few	Wilcoxon signed-rank test	0.001	0.9313
BertScore	Zero	Wilcoxon signed-rank test	0.0393	0.2244
BertScore	Few	Wilcoxon signed-rank test	0.0026	≤ 0.001

**Table 5:** The impact of employing different prompts on the performance of language models in text generation tasks. The table includes results from *t*-paired tests and Wilcoxon signed-rank tests applied to ROUGE-1, ROUGE-2, ROUGE-L, Instructor Similarity, and BERTScore metrics, evaluating the consistency and accuracy of the models' outputs.

**Metric**	**Prompt**	**Test type**	**Lilliefors ** **p** ** value**	**p** ** value**
ROUGE–1	Prompt1	*t*–paired test	0.5717	0.0131
ROUGE–1	Prompt 2	*t*-paired test	0.1244	0.0877
ROUGE–2	Prompt 1	Wilcoxon signed-rank test	0.0118	0.1196
ROUGE –2	Prompt 2	Wilcoxon signed-rank test	0.006	0.8482
ROUGE–L	Prompt 1	*t*-paired test	0.2865	0.0446
ROUGE–L	Prompt 2	*t*-paired test	0.3506	0.7022
Instructor Similarity	Prompt 1	Wilcoxon signed-rank test	0.0262	0.5411
Instructor Similarity	Prompt 2	Wilcoxon signed-rank test	0.001	0.014
BERTScore	Prompt 1	*t*-paired test	0.8653	0.6845
BERTScore	Prompt 2	*t*-paired test	0.2054	≤ 0.001

## Data Availability

The data utilized in this study are confidential in nature and cannot be shared due to privacy and confidentiality agreements but are available from the corresponding author upon reasonable request. However, the code implemented in the analysis is openly available in a GitHub repository https://github.com/aidikhcc/RadiologyNLP. Researchers interested in replicating or extending this work are encouraged to access the codebase and adapt it to the local dataset.
